# Proliferation of MDSCs may indicate a lower ^CD4+^ T cell immune response in schistosomiasis japonica[Fn FN1]

**DOI:** 10.1051/parasite/2024050

**Published:** 2024-08-29

**Authors:** Bo Peng, Yulin Luo, Shudong Xie, Quan Zhuang, Junhui Li, Pengpeng Zhang, Kai Liu, Yu Zhang, Chen Zhou, Chen Guo, Zhaoqin Zhou, Jie Zhou, Yu Cai, Meng Xia, Ke Cheng, Yingzi Ming

**Affiliations:** 1 Transplantation Center, The Third Xiangya Hospital, Central South University Changsha Hunan China; 2 NHC Key Laboratory of Translational Research on Transplantation Medicine Changsha Hunan China; 3 Schistosomiasis Control Institute of Hunan Province Yueyang Hunan China; 4 Xiangyue Hospital affiliated to Hunan Institute of Schistosomiasis Control Yueyang Hunan China

**Keywords:** *Schistosoma japonicum*, Granulocytic myeloid-derived suppressor cells, Liver fibrosis

## Abstract

*Background: Schistosoma japonicum* (*S. japonicum*) is the main species of *Schistosoma* prevalent in China. Myeloid-derived suppressor cells (MDSCs) are important immunoregulatory cells and generally expand in parasite infection, but there is little research relating to MDSCs in *Schistosoma* infection. *Methods:* Fifty-six *S. japonicum*-infected patients were included in this study. MDSCs and percentages and absolute cell numbers of lymphocyte subsets, including ^CD3+^ T cells, ^CD4+^ T cells, ^CD8+^ T cells, B cells and natural killer (NK) cells were detected using flow cytometry. The degree of liver fibrosis was determined using color Doppler ultrasound. *Results:* Patients infected with *S. japonicum* had a much higher percentage of MDSCs among peripheral blood mononuclear cells (PBMCs) than the healthy control. Regarding subpopulations of MDSCs, the percentage of granulocytic myeloid-derived suppressor cells (G-MDSCs) was clearly increased. Correlation analysis showed that the absolute cell counts of T-cell subsets correlated negatively with the percentages of MDSCs and G-MDSCs among PBMCs. The percentage of G-MDSCs in PBMCs was also significantly higher in patients with liver fibrosis diagnosed by color doppler ultrasound (grade > 0), and the percentage of G-MDSCs in PBMCs and liver fibrosis grading based on ultrasound showed a positive correlation. *Conclusion: S. japonicum* infection contributes to an increase in MDSCs, especially G-MDSCs, whose proliferation may inhibit the number of ^CD4+^ T cells in peripheral blood. Meanwhile, there is a close relationship between proliferation of G-MDSCs and liver fibrosis in *S. japonicum*-infected patients.

## Abstract (fr)

*Contexte : Schistosoma japonicum* est la principale espèce de *Schistosoma* répandue en Chine. Les cellules myéloïdes suppressives (MDSC) sont des cellules immunorégulatrices importantes et se développent généralement lors d’une infection parasitaire, mais il existe peu de recherches sur les MDSC dans l’infection à *Schistosoma*. *Méthodes :* Cinquante-six patients infectés par *S. japonicum* ont été inclus dans cette étude. Les MDSC, les pourcentages et les nombres absolus des sous-ensembles de lymphocytes, notamment les lymphocytes T ^CD3+^, les lymphocytes T ^CD4+^, les lymphocytes T ^CD8+^, les lymphocytes B et les cellules tueuses naturelles (NK) ont été détectés par cytométrie en flux. Le degré de fibrose hépatique a été déterminé par échographie Doppler couleur. *Résultats :* Les patients infectés par *S. japonicum* présentaient un pourcentage beaucoup plus élevé de MDSC parmi les cellules mononucléées du sang périphérique (CMSP) que les patients sains. En ce qui concerne les sous-populations de MDSC, le pourcentage de cellules suppressives granulocytaires dérivées de myéloïdes (G-MDSC) était augmenté de manière évidente. L’analyse de corrélation a montré que le nombre absolu des cellules des sous-ensembles de lymphocytes T était en corrélation négative avec les pourcentages de MDSC et de G-MDSC parmi les CMSP. Le pourcentage de G-MDSC dans les CMSP était également significativement plus élevé chez les patients présentant une fibrose hépatique diagnostiquée par échographie Doppler couleur (grade > 0), et le pourcentage de G-MDSC dans les CMSP et le classement de la fibrose hépatique basé sur l’échographie ont montré une corrélation positive. *Conclusion :* L’infection à *S. japonicum* contribue à une augmentation des MDSC, notamment des G-MDSC, dont la prolifération pourrait inhiber le nombre de lymphocytes T ^CD4+^ dans le sang périphérique. Parallèlement, il existe une relation étroite entre la prolifération des G-MDSC et la fibrose hépatique chez les patients infectés par *S. japonicum*.

## Introduction

It is estimated that more than 250 million people in 78 countries around the world are affected by schistosomiasis, causing approximately 280,000 deaths annually [22]. At present, there are six known species of *Schistosoma* that infect humans, but only *Schistosoma japonicum* (*S. japonicum*) is endemic in China. Several studies have confirmed that substances from *S. japonicum* have immune regulatory functions, such as promotion of M2 macrophage polarization by soluble egg antigen (SEA) [44], inhibition of cytokine production of proinflammatory Th1, Th2 and Th17 cells by recombinant Sj16 protein [41], suppression of exogenous antigen presentation of dendritic cells by recombinant cysteine protease inhibitor [2], and inhibition of lipopolysaccharide-stimulated dendritic cell (DC) maturation and induction of M2-type polarization of RAW264.7 macrophages by recombinant Sj CP1412 [18].

In healthy people, immature marrow cells (IMCs) in bone marrow can rapidly differentiate into mature granulocytes, macrophages or DC, and various pathological conditions, such as infection, cancer, bone marrow transplantation and autoimmune diseases, interfere with their differentiation, resulting in accumulation of myeloid-derived suppressor cells (MDSCs) [10]. MDSCs are characterized by T-cell immunosuppressive functions [13], which inhibit T cells through arginase-1 [17] and reactive oxygen species (ROS) production [31] or inhibit T-cell activation by downregulating L-selectin expression on naïve T cells [29]. In general, human MDSCs are classified into granulocytic myeloid-derived suppressor cells (G-MDSCs) (CD11b^+^CD14^-^CD15^+^) and monocytic myeloid-derived suppressor cells (M-MDSCs) (CD11b^+^CD14^+^HLA-DR^low/-^CD15^-^) [33] after gradient density centrifugation from peripheral blood. Although other markers of MDSCs, including CD80 (also known as B7.1) [52], CD115 (also known as the macrophage colony stimulating factor receptor) [8, 15], and CD124 (also known as IL-4 receptor α chain) [8], are reported to recognize MDSCs, they might not participate in the immunosuppressive function of MDSCs [26, 53].

As an important member of the immune regulatory network, MDSCs have recently been found to proliferate in mice infected with *S. japonicum* [55, 60], but there is little research on human parasitic infections. Therefore, we detected the distribution of MDSCs and lymphocyte subsets in the peripheral blood of patients infected with *S. japonicum*. The findings provide a new perspective on interaction between immune cells and MDSCs induced by human parasite infection.

## Materials and methods

### Ethics approval and consent to participate

The studies involving human participants were reviewed and approved by the Institutional Review Board of Third Xiangya Hospital, Central South University. The patients/participants provided their written informed consent to participate in this study.

### Inclusion and exclusion criteria for the study population

This was a retrospective case-control study on *S. japonicum* infection. The patients enrolled were from Schistosomiasis Control Hospitals in Changde and Yueyang, Hunan Province, China. The two hospitals mainly treat patients infected by parasites from Dongting Lake. The inclusion criteria of this study included the follows: (i) epidemiological history involving a history of contact with schistosome-inhabited water or a history of schistosome infection and at least one praziquantel treatment; (ii) previous diagnosis of schistosomiasis and hospitalization; (iii) clinical symptoms or signs, including fatigue, mucous stool, splenomegaly, ascites, etc.; and (iv) any positive result of schistosome IgG antibodies or schistosome eggs in stool, or abnormal liver function or coagulation function, or typical imaging findings of schistosomiasis cirrhosis by liver ultrasound or liver CT. In addition, 21 healthy people were included as the healthy controls (HCs) group. Basic demographic information is shown in Supplementary Table 1.

The exclusion criteria of patients with *S. japonicum* infection were as follows: (i) schistosomiasis with viral hepatitis (including positive results of hepatitis B surface antigen, hepatitis B DNA and hepatitis C antibody), alcoholic hepatitis, or metabolic dysfunction-associated fatty liver disease (MAFLD); (ii) incompleteness of MDSC and lymphocyte subgroup data; (iii) liver cirrhosis without *S. japonicum* infection; (iv) combined specific infections other than schistosomiasis, such as sepsis, bacteremia, human immunodeficiency virus (HIV), and syphilis; (v) younger than 18 years old; (vi) pregnancy; and (vii) liver cancer, gastric cancer, colorectal cancer and other malignant tumors complicated with schistosomiasis.

### B-ultrasound grading

According to Niamey Ultrasound Guide in Schistosomiasis [6], degrees of liver fibrosis in those infected with *S. japonicum* were divided into four grades: grade 0, grade I, grade II and grade III [14]. Grade 0 was defined as the grade = 0 group; grades I, II and III were defined as the grade > 0 group. The splenic thickness, length of the spleen under the ribs, inner diameter of the hepatic portal vein, depth of abdominal dropsy, long diameter of the left lobe of the liver, and anterior and posterior diameter of the left hepatic lobe and maximum oblique diameter of the right lobe of the liver were measured by ultrasound. Splenectomy, the degree of liver cirrhosis, portal hypertension, cavernous change and thrombosis of the portal vein were also assessed.

### Flow cytometry analysis of peripheral blood

**Supplementary Figure 1:**
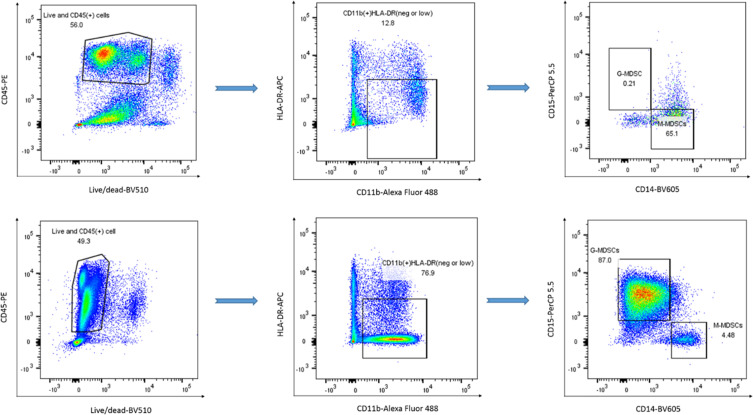
Flow cytometry gate strategy for MDSCs in healthy individuals and patients infected with *Schistosoma japonicum*. CD45^+^ and live cells are gated from PBMCs through CD45-PE and live/dead dyes-BV510. Next, CD11b^+^HLA-DR^-/low^ cells are gated through CD11b-Alexa Fluor 488 and HLA-DR-APC. Finally, through CD14-BV605 and CD15-PerCP 5.5, CD11b^+^HLA-DR^-/low^CD14^+^ M-MDSC and CD11b^+^HLA-DR^-/low^CD15^+^ G-MDSCs are gated, respectively. The first row shows the distribution of G-MDSCs and M-MDSCs in the peripheral blood of healthy individuals, while the second row shows the distribution of G-MDSCs and M-MDSCs in the peripheral blood of patients infected with *Schistosoma japonicum*.

The MDSC [33] panel and TBNK [32] panel were used as described in our previous work. Briefly, a fresh ethylene diamine tetraacetic acid (EDTA)-treated peripheral blood sample, approximately 3 mL in volume, was fully mixed, and then 50 μL was added to an absolute cell counting tube containing counting microspheres. Ten microliters of BD Multitest 6-color TBNK reagent was added and incubated at room temperature in the dark for 15 minutes. Then, 450 μL of red blood cell lysate was added, swirled and mixed, and detected by flow cytometry after 10 minutes. The remaining sample was diluted in equal proportions with room temperature Dulbecco’s phosphate-buffered saline (DPBS), which was carefully added to an appropriate amount of Ficoll solution (1.077 g/mL, Sigma Aldrich, St. Louis, MO, USA) and then centrifuged at room temperature 400 x *g* without acceleration and braking for 30 minutes. Afterward, PBMCs were extracted from the buffy coat and stained for the MDSC panel (Supplementary Figure 1) at 4 ℃ for 30 min in the dark after incubation with live/dead dye (Zombie Aqua^TM^ Fixable Viability Kit, Cat: 423101, BioLegend, San Diego, CA, USA). Flow cytometry staining buffer (REF: 00-4222-26, eBioscience, San Diego, CA, USA) was used to wash the cells twice after MDSC staining, and the cell suspension was detected by flow cytometry. Flow Jo 10.8 software was used for data analysis.

### Statistical analysis

Graph Pad Prism version 7.0 was used for mapping and statistical analysis. Continuous numerical variables are represented by the mean ± standard deviation. Differences between two groups of continuous numerical variables were compared using unpaired *t* tests. Fisher’s exact test or the chi-square test was applied to compare rates for the two groups. When the rate for two groups was close to 0 or 1, Fisher’s exact test was performed. The Mann–Whitney test was used to compare the range and median of two groups of variables. Pearson correlation analysis was employed to analyze correlation between two groups of continuous variables, with r expressing the degree of correlation. A value closer to 1 or −1 indicated a greater correlation between two groups; when the value was closer to 0, no correlation between two groups was considered. A bilateral confidence interval ≥95% and a *p* value < 0.05 indicated a significant difference.

## Results

### Population characteristics

**Figure 1:**
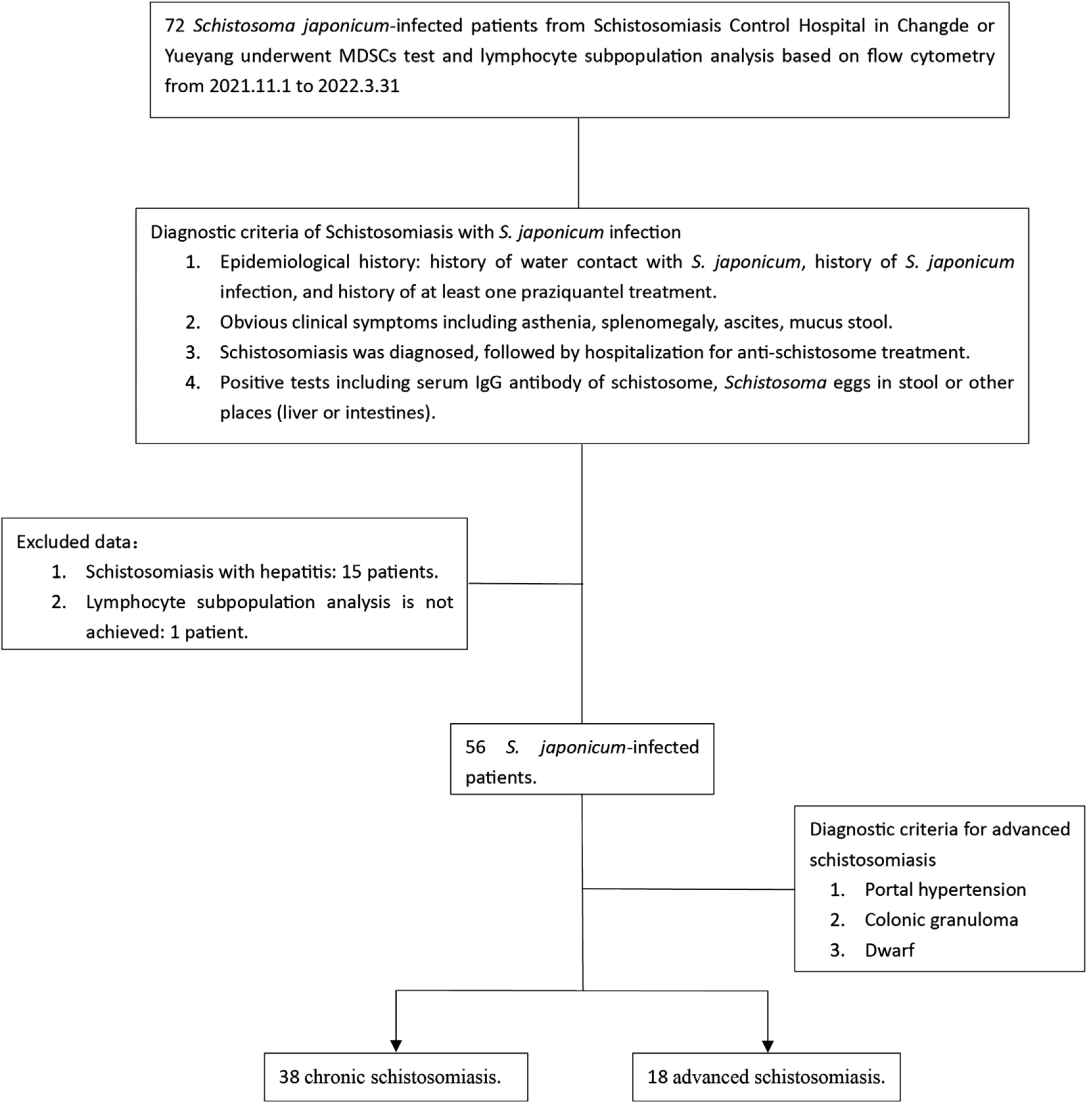
Study flow chart. *S. japonicum, Schistosoma japonicum*. MDSCs, myeloid-derived suppressor cells.

From November 1, 2021, to March 31, 2022, a total of 72 patients with *S. japonicum* infection were admitted from Schistosomiasis Control Hospital in Changde or Yueyang. Following the diagnostic criteria of schistosomiasis [24, 56] as well as the inclusion and exclusion criteria of this study (Figure 1), 56 patients with *S. japonicum* infection were included: 38 clinically diagnosed with chronic schistosomiasis japonica (CSJ) and 18 clinically diagnosed with advanced schistosomiasis japonica (ASJ). Fifteen patients with viral hepatitis were excluded, 8 of whom had advanced schistosomiasis (6 of whom were hepatitis B patients and 2 of whom were hepatitis C patients); 7 patients had chronic schistosomiasis (6 of whom were hepatitis B patients and 1 of whom had hepatitis C). One patient without lymphocyte subpopulation analysis was excluded.

Among *S. japonicum* patients, 45 underwent B-ultrasound examination, with 29 in the CSJ group and 16 in the ASJ group. A total of 41.38% of patients with CSJ were categorized into the grade 0 group, whereas grade III was primarily diagnosed in the ASJ group (Table 1). In the ASJ group, 3 patients experienced portal hypertension: 1 case of esophageal gastric varices under gastroscopy and 2 cases of portal hypertension diagnosed by ultrasound. Compared with the CSJ group, the ASJ group had higher values of splenic thickness, length of the spleen under the ribs, splenectomy rate, inner diameter of the hepatic portal vein, depth of abdominal dropsy, liver cirrhosis rate, and portal hypertension rate (*p* < 0.05). There were no differences in the long diameter of the left lobe of the liver, anterior and posterior diameter of the left hepatic lobe, maximum oblique diameter of the right lobe of the liver, rate of cavernous change or thrombosis of the portal vein (Supplementary Table 2).

**Table 1:** Clinical data of patients infected with *S. japonicum*.

Clinical characteristics	All patients, *n* = 56	CSJ, *n* = 38	ASJ, *n* = 18	*p* value^∇^
Male, *n* (%)	39 (69.64%)	26 (68.42%)	13 (72.22%)	0.773^ζ^
Age (Mean ± SD, y)	60.57 ± 11.21	59.50 ± 10.48	62.83 ± 12.63	0.303^†^
Grade, *n* (%)				<0.001^ζ^
Grade 0	12 (26.67%)	12 (41.38%)	0 (0.00%)	
Grade I	11 (24.44%)	11 (37.93%)	0 (0.00%)	
Grade II	6 (13.33%)	5 (17.24%)	1 (6.25%)	
Grade III	16 (35.56%)	1 (3.45%)	15 (93.75%)	
Monocyte count (Mean ± SD) (10^9^/L)	0.40 ± 0.23	0.34 ± 0.12	0.51 ± 0.34	0.024^†^
Percentage of monocytes among leukocytes (Mean ± SD) (%)	6.87 ± 2.72	5.99 ± 1.74	8.51 ± 3.47	0.003^†^
Total bile acid (TBA, μmMol/L) (Mean ± SD)	8.01 ± 11.06	4.61 ± 2.51	15.80 ± 17.74	0.001^†^
Aspartate aminotransferase (AST, U/L) (Mean ± SD)	27.22 ± 9.81	25.09 ± 6.40	32.07 ± 14.11	0.025^†^
Albumin (ALB, g/L) (Mean ± SD)	44.91 ± 6.51	47.03 ± 4.31	40.05 ± 8.13	<0.001^†^
Activated partial prothrombin time (aPTT, Sec) (Mean ± SD)	25.08 ± 6.82	22.65 ± 3.07	27.87 ± 8.83	0.041^†^
Prothrombin time (PT, Sec) (Mean ± SD)	10.89 ± 2.10	10.17 ± 1.07	11.73 ± 2.68	0.047^†^
Schistosome eggs, *n* (%)	0 (0.00%)	0 (0.00%)	0 (0.00%)	> 0.999^╜^
Schistosome IgG antibodies, *n*/*N* (%)	17/42 (40.48%)	15/30 (50.00%)	2/12 (16.67%)	0.047^ζ^

∇: Comparison between patients with CSJ and ASJ. ζ: Tested by Chi-square test. †: Tested by Unpaired t test. ╜: Tested by Fisher’s exact test. К: Tested by Mann–Whitney test. *S. japonicum, Schistosoma japonicum*. CSJ, chronic schistosomiasis japonica. ASJ, advanced schistosomiasis japonica. SD, standard deviation.

Serum indicators showing significant differences between the CSJ and ASJ groups were total bile acid (TBA), aspartate aminotransferase (AST), albumin (ALB) and schistosome IgG antibody, but there was no significant difference in other serum indicators, such as γ-glutamyl transpeptidase (γ-GT), total bilirubin concentration (TBIL), direct bilirubin concentration (DBIL), alanine aminotransferase concentration (ALT), and globulin (GLO). The count and percentage of monocytes in the peripheral white blood cells were significantly increased in the ASJ group, with hemoglobin (HGB) and count levels of eosinophils, neutrophils, lymphocytes and platelets similar to those of the CSJ group. Differences in coagulation function indices were mainly reflected in the activated partial prothrombin time (aPTT) and prothrombin time (PT) but not in fibrinogen (FIB) or the international normalized ratio (INR) (Table 1 and Supplementary Table 2). It is worth noting that in this study, no schistosome eggs were found in the stool of any *S. japonicum-*infected patient. In addition, 15 of 30 CSJ patients had positive schistosome IgG antibodies, while only 2 of 12 ASJ patients were positive (Table 1).

### Significant increase of MDSCs and G-MDSCs in *S. japonicum-*infected patients

**Figure 2:**
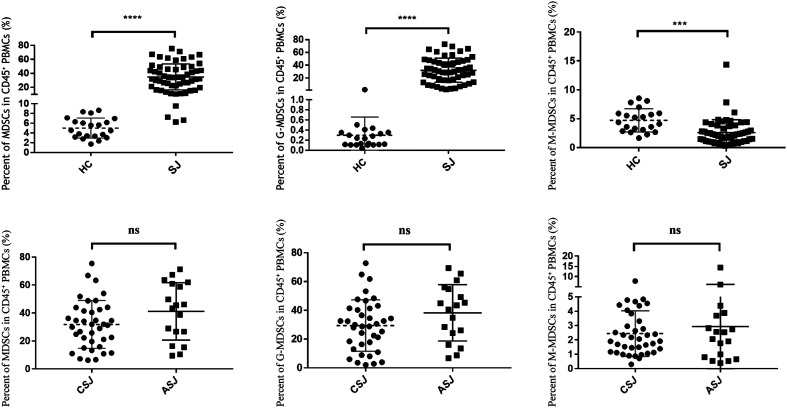
Distribution of MDSCs in peripheral blood. (A) Percent of MDSCs, G-MDSCs, M-MDSCs among CD45^+^ PBMCs of HC and SJ groups. (B) Percent of MDSCs, G-MDSCs, M-MDSCs among CD45^+^ PBMCs of CSJ and ASJ groups. MDSCs, myeloid-derived suppressor cells. G-MDSCs, granulocytic MDSCs. M-MDSCs, monocytic MDSCs. PBMCs, peripheral blood mononuclear cells. HC, healthy control. SJ, schistosomiasis japonica. CSJ, chronic schistosomiasis japonica. ASJ, advanced schistosomiasis japonica.

The percentage of MDSCs and the two subgroups in PBMCs were compared between the HC group and schistosomiasis japonica (SJ) group. As shown in Figure 2, MDSC levels were much higher in the SJ group than in the HC group (34.85% ± 18.61% vs. 5.03% ± 2.07%, *p* < 0.001). Regarding subsets of MDSCs, the G-MDSC subset was much higher in the SJ group (32.25% ± 18.76% vs. 0.30% ± 0.36%, *p* < 0.001) and the M-MDSC subset was lower in the SJ group (2.60% ± 2.24 % vs. 4.73% ± 2.01%, *p* < 0.001). In a subgroup analysis of the SJ group, the CSJ group had a lower MDSC level compared with the ASJ group (31.84% ± 17.11% vs. 41.20% ± 20.51%, *p* = 0.079), as well as G-MDSCs (29.39% ± 17.92% vs. 38.27% ± 19.57%, *p* = 0.099) and M-MDSCs (2.44% ± 1.59% vs. 2.93% ± 3.25%, *p* = 0.454), but without significance (Table 2, Figure 2).

**Table 2:** Distribution of MDSCs and lymphocyte subsets in *S. japonicum*-infected patients and healthy controls.

	All persons, *n* = 77	SJ, *n* = 56			Healthy controls, *n* = 21	*p* value^◇^
		CSJ, *n* = 38	ASJ, *n* = 18	*p* value^▽^		
Percent of MDSCs among CD45^+^ PBMCs (Mean ± SD) (%)	26.71 ± 20.75	34.85 ± 18.61			5.03 ± 2.07	<0.001
		31.84 ± 17.11	41.20 ± 20.51	0.079		
Percent of G-MDSCs among CD45^+^ PBMCs (Mean ± SD) (%)	23.53 ± 21.44	32.25 ± 18.76			0.30 ± 0.36	<0.001
		29.39 ± 17.92	38.27 ± 19.57	0.099		
Percent of M-MDSCs among CD45^+^ PBMCs (Mean ± SD) (%)	3.18 ± 2.37	2.60 ± 2.24			4.73 ± 2.01	<0.001
		2.44 ± 1.59	2.93 ± 3.25	0.454		
Absolute cell counts of CD3^+^ cells (Mean ± SD) (10^9^/L)	1.08 ± 0.44	1.07 ± 0.48			1.13 ± 0.35	0.583
		1.12 ± 0.40	0.95 ± 0.60	0.199		
Absolute cell counts of CD3^+^ CD4^+^ cells (Mean ± SD) (10^9^/L)	0.59 ± 0.22	0.59 ± 0.23			0.59 ± 0.20	0.997
		0.63 ± 0.21	0.50 ± 0.25	0.051		
Absolute cell counts of CD3^+^ CD8^+^ cells (Mean ± SD) (10^9^/L)	0.43 ± 0.26	0.41 ± 0.30			0.46 ± 0.15	0.496
		0.41 ± 0.25	0.41 ± 0.38	0.963		
Absolute cell counts of CD19^+^ cells (Mean ± SD) (10^9^/L)	0.19 ± 0.11	0.19 ± 0.11			0.19 ± 0.12	0.734
		0.19 ± 0.09	0.18 ± 0.13	0.776		
Absolute cell counts of CD16^+^ CD56^+^ cells (Mean ± SD) (10^9^/L)	0.31 ± 0.22	0.35 ± 0.25			0.22 ± 0.09	0.023
		0.37 ± 0.26	0.29 ± 0.21	0.271		

▽: Comparison between patients with CSJ and ASJ; ◇: Comparison between healthy controls and *S. japonicum*-infected patients.

MDSCs, myeloid-derived suppressor cells. *S. japonicum*, Schistosoma japonicum. SJ, schistosomiasis japonica. CSJ, chronic schistosomiasis japonica. ASJ, advanced schistosomiasis japonica. PBMCs, peripheral blood mononuclear cells. SD, standard deviation. G-MDSCs, granulocytic myeloid-derived suppressor cells. M-MDSCs, monocytic myeloid-derived suppressor cells.

### Absolute cell counts of T-cell subsets were negatively correlated with percentages of MDSCs and G-MDSCs in PBMCs

Absolute cell counts of T cells, B cells, and NK cells were compared between the HC group and SJ group. As shown in Table 2, T cells were not obviously lower in the SJ group than in the HC group (1.07 × 10^9^/L ± 0.48 × 10^9^/L vs. 1.13 × 10^9^/L ± 0.35 × 10^9^/L, *p* = 0.583), and B cells were almost at the same level (0.19 × 10^9^/L ± 0.11 × 10^9^/L vs. 0.19 × 10^9^/L ± 0.12 × 10^9^/L, *p* = 0.734). However, NK cells were present at much higher levels in the SJ group than in the HC group (0.35 × 10^9^/L ± 0.25 × 10^9^/L vs. 0.22 × 10^9^/L ± 0.09 × 10^9^/L, *p* = 0.023). Consequently, subgroup analysis was carried out on the SJ group. As shown in Table 2, no significant differences in T cells, B cells and NK cells were found between the CSJ and ASJ groups. With respect to subsets of T cells, there were no differences in CD4^+^ T cells (0.50 × 10^9^/L ± 0.25 × 10^9^/L vs. 0.63 × 10^9^/L ± 0.21 × 10^9^/L, *p* = 0.051) or CD8^+^ T cells (0.41 × 10^9^/L ± 0.38 × 10^9^/L vs. 0.41 × 10^9^/L ± 0.25 × 10^9^/L, *p* = 0.963).

**Figure 3:**
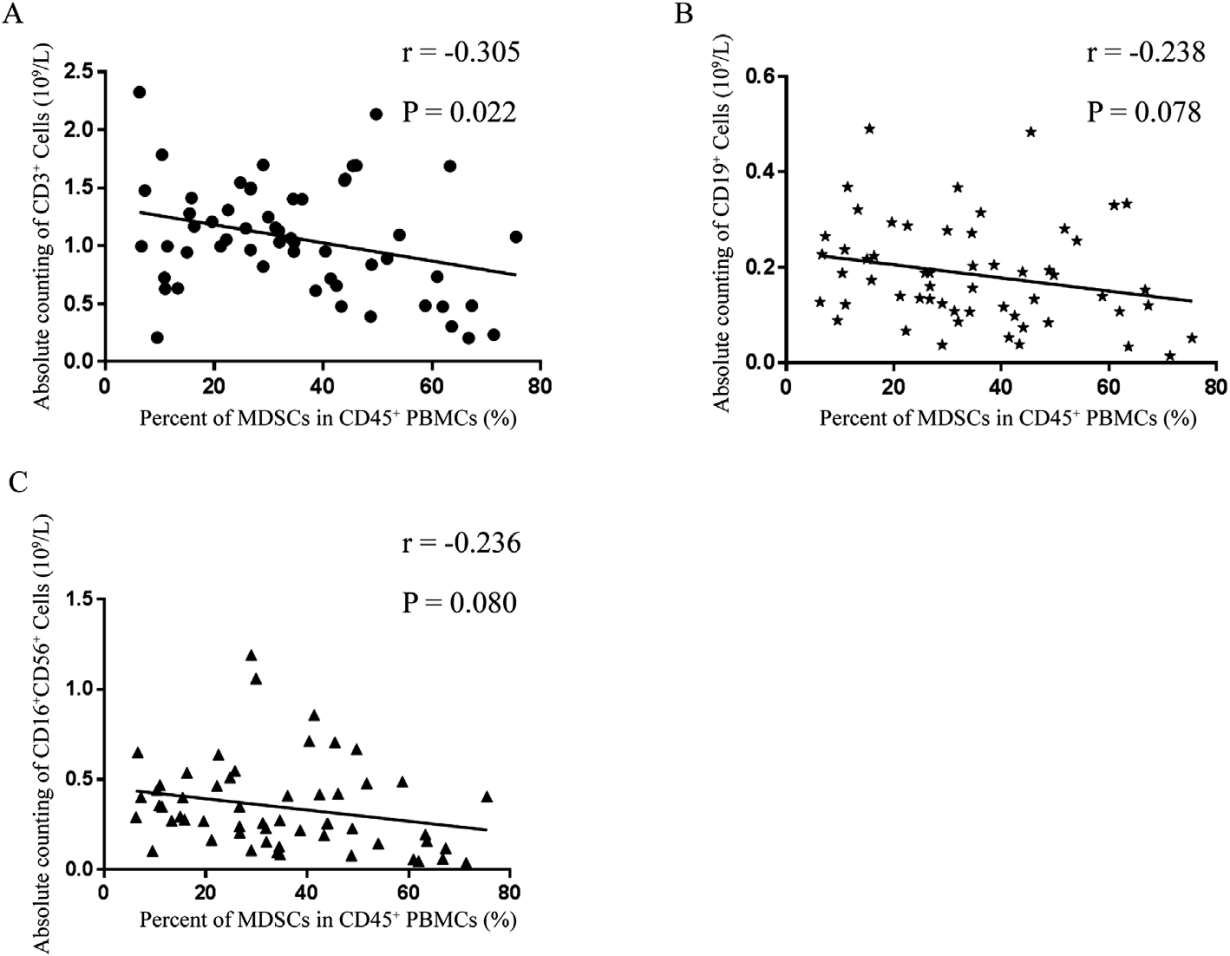
Correction between absolute cell counts of lymphocyte subsets and MDSCs among PBMCs of SJ patients. (A) CD3^+^ T cells and MDSCs. (B) CD19^+^ B cells and MDSCs. C, CD16^+^CD56^+^ NK cells and MDSCs. MDSCs, myeloid-derived suppressor cells. PBMCs, peripheral blood mononuclear cells. SJ, schistosomiasis japonica. NK, natural killer.

**Figure 4:**
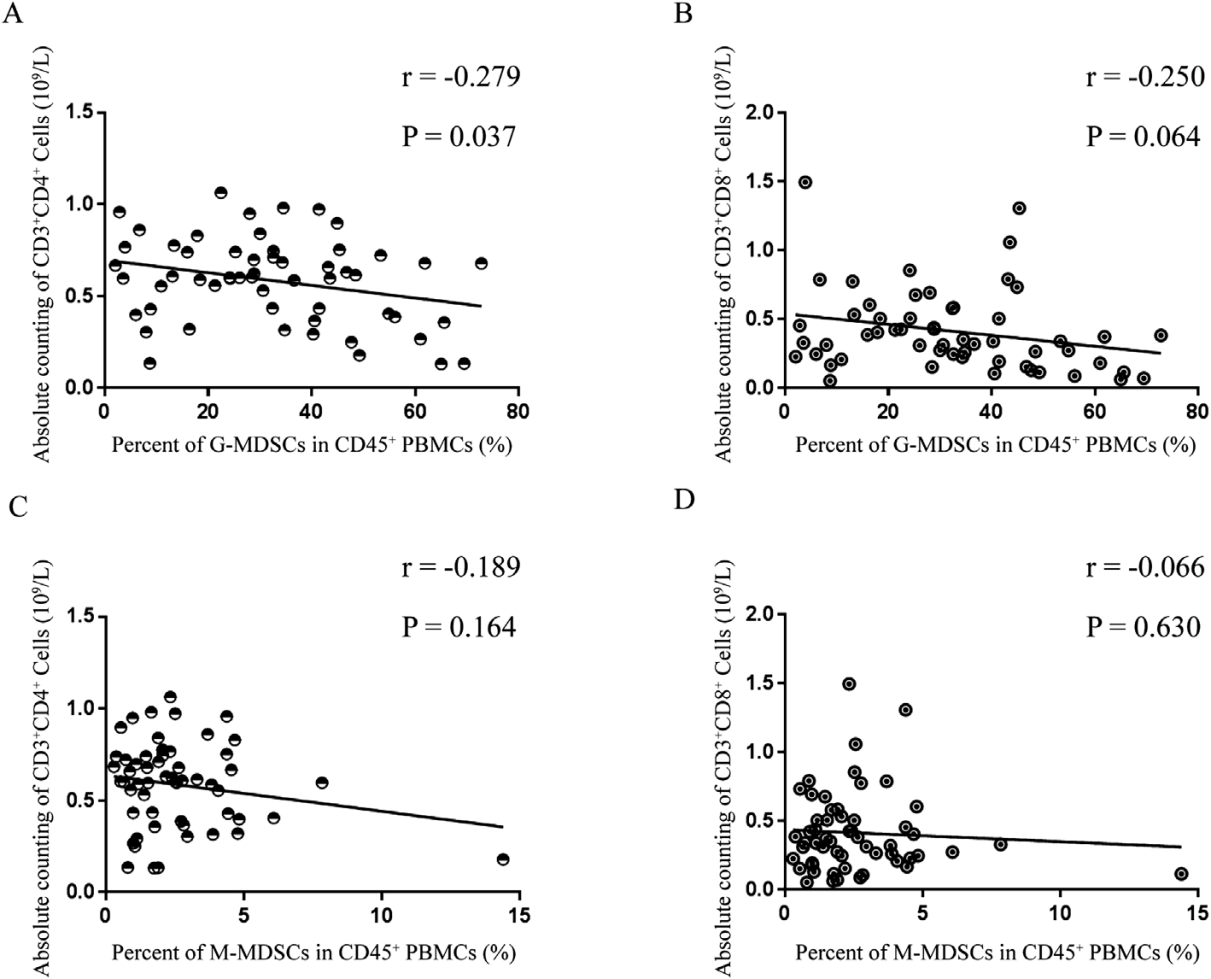
Correction between absolute cell counts of T lymphocyte subsets and MDSC subsets among PBMCs of SJ patients. (A) CD3^+^CD4^+^ T cells and G-MDSCs. (B) CD3^+^CD8^+^ T cells and G-MDSCs. C, CD3^+^CD4^+^ T cells and M-MDSCs. D, CD3^+^CD8^+^ T cells and M-MDSCs. G-MDSCs, granulocytic MDSCs. M-MDSCs, monocytic MDSCs. PBMCs, peripheral blood mononuclear cells. SJ, schistosomiasis japonica.

As shown in Figure 3, correlation analysis between the percentage of MDSCs and the absolute cell counts of T cells, B cells or NK cells was performed; the percentage of MDSCs and the absolute cell count of T cells had the highest correlation (*r* = −0.305, *p* = 0.022), though no significant difference between the percentage of MDSCs and absolute cell count of B cells (*r* = −0.238, *p* = 0.078) or NK cells (*r* = −0.236, *p* = 0.080) was found. T-cell subsets and MDSC subsets were further analyzed for correlations. The absolute cell count of CD4^+^ T cells and percentage of G-MDSCs had the highest correlation (*r* = −0.279, *p* = 0.037), but there was no correlation for absolute cell count of CD8^+^ T cells and percentage of G-MDSCs (*r* = −0.250, *p* = 0.064), absolute cell count of CD4^+^ T cells and percentage of M-MDSCs (*r* = −0.189, *p* = 0.164), or absolute cell count of CD8^+^ T cells and percentage of M-MDSCs (*r* = −0.066, *p* = 0.630) (Figure 4).

### G-MDSCs clearly accumulated in patients with liver fibrosis induced by *S. japonicum* infection

**Figure 5:**
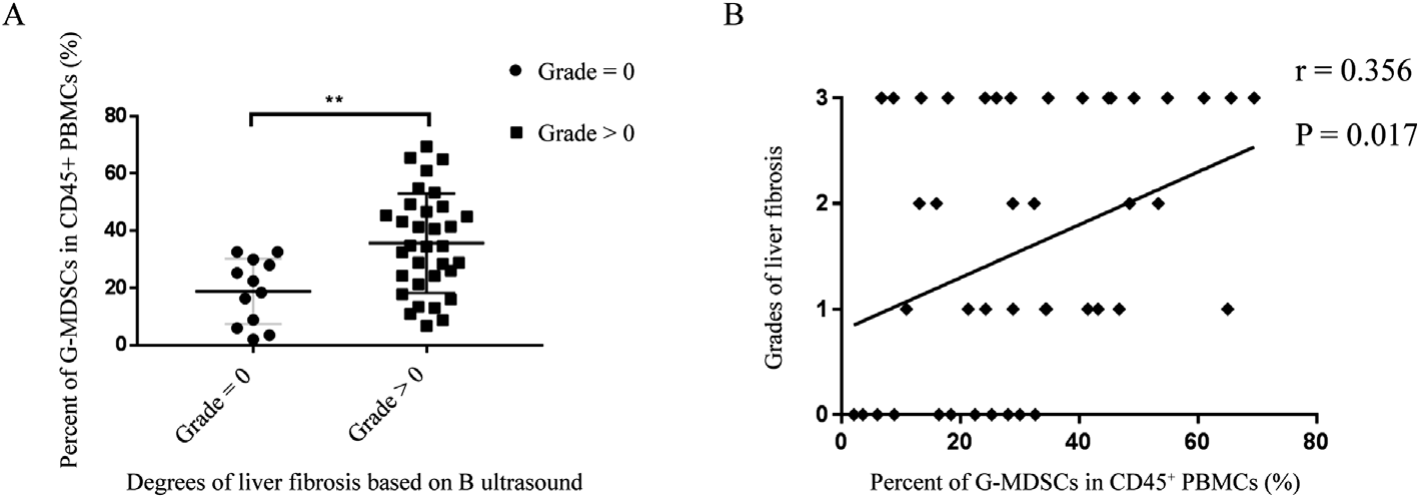
G-MDSCs accumulated in SJ patients with liver fibrosis. (A) Percent of G-MDSCs among PBMCs (%) in grade = 0 and grade > 0 groups. (B) Correction between percent of G-MDSCs among PBMCs (%) and degrees of liver fibrosis based on B ultrasound in SJ patients. G-MDSCs, granulocytic MDSCs. PBMCs, peripheral blood mononuclear cells. SJ, schistosomiasis japonica.

Among 56 patients infected with *S. japonicum*, B-ultrasound images for rating were available for 45, and 12 patients were in the grade = 0 group, all of whom were in the CSJ group (Table 1). There were 33 patients in the grade > 0 group, 16 of whom were from the ASJ group and 17 from the CSJ group. The analysis showed that the percentage of G-MDSCs in the grade > 0 group was significantly higher than that in the grade = 0 group (35.61% ± 17.34%vs. 18.86% ± 11.39%, *p* = 0.003) (Figure 5A). As illustrated in Figure 5B, correlation analysis between the percentage of G-MDSCs among PBMCs and liver fibrosis grade based on ultrasound was performed, with a positive correlation found (*r* = 0.356, *p* = 0.017).

## Discussion

MDSC proliferation may occur in infectious diseases due to a variety of pathogens, including bacteria, fungi and chronic hepatitis C virus or HIV [47], as well as mouse parasitic infection models (such as *Schistosoma*, *Plasmodium*, *Leishmania*, *Taenia crassiceps*, and *Brugia malayi*) [43, 46]. At present, there is some evidence that MDSCs expand in the peripheral blood of patients with sepsis as a consequence of altered myelopoiesis, which has led to a novel approach to target MDSCs and their immunosuppressive function [48].

In sepsis, G-MDSCs (also known as PMN-MDSCs, polymorphonuclear myeloid-derived suppressor cells) expand mainly in response to gram-positive pathogens, whereas M-MDSC expansion is caused by both gram-positive and gram-negative pathogens [16]. In the early stages of sepsis, MDSCs are probably present at very low frequency, which protects the host and leads to a better outcome [35]. However, if the infection is not resolved, MDSCs increase and exert a sustained T-cell immunosuppressive effect, leading to a worse outcome [45]. Therefore, evaluating the distribution of MDSCs and their subtypes in peripheral blood is helpful to judge the disease outcome of patients with sepsis.

Accumulation of MDSCs also occurs in mice infected with schistosomes, whose products, such as SEA and schistosome worm antigen (SWA), induce MDSCs *in vitro* [51]. However, when humans are infected with schistosomes, the frequency of MDSCs is not clear. Yet clues may be obtained from clinical trials of other parasitic infections in humans.

Recently, one study described the distribution of MDSCs in peripheral blood during *Plasmodium falciparum* infection: G-MDSCs (CD11b^+^CD33^+^HLA-DR^+/lo^CD14^-^) mainly proliferated and were related to a decrease in peripheral blood lymphocytes. *In vitro* experiments confirmed that G-MDSCs reduce the proliferation rate of T cells by 50% but that M-MDSCs (CD11b^+^CD33^+^HLA-DR^+/lo^CD14^+^) have no obvious correlation with the number of Tregs in the blood circulation. Therefore, it was proposed that G-MDSCs might be used as an early diagnostic indicator of plasmodium infection, and preventive antimalarial treatment should be carried out for vaccinees to reverse malaria-related G-MDSC immunosuppression, improving the response of exposed individuals to the vaccine [19]. In the latest study on human infection with malaria parasites (*Plasmodium falciparum* and *Plasmodium vivax*), it was again confirmed that the absolute number of G-MDSCs (SCC^hi^CD15^+^CD66b^+^CD11b^+^CD14^-^) increase in peripheral blood [20].

Normally, there is a very low proportion of G-MDSCs in healthy people, accounting for 0.1 ~ 1% of PBMCs [1, 39]. Based on the HC population from different communities, the highest proportion of G-MDSCs does not exceed 5%. In our study, the average percentage of G-MDSCs in healthy people was 0.301%. However, G-MDSCs accumulate and restrict immune responses in a variety of diseases, such as multiple myeloma [34], late sepsis [3], COVID-19 [4], malaria infection [19], and non-small cell lung cancer with LKB1-inactivating mutations [21]. G-MDSCs and neutrophils share an origin and morphological and phenotypic features. Accumulating evidence indicates that G-MDSCs are pathologically activated neutrophils [59].

We found that the percentage of G-MDSCs in PBMCs correlated positively with the severity of schistosomiasis liver fibrosis, which might be related to immune escape of parasites by negative regulation of the immune network in infected individuals. For instance, *Schistosoma mansoni* (*S. mansoni*) SmKI-1 interferes with neutrophil migration and function by inhibiting the activity of neutrophil elastase, resulting in a decline in the capacity to kill the schistosomes [25]. Two DNases have been identified to hydrolyze neutrophil extracellular traps (NETs) in African trypanosomatid parasites, counteracting host innate immune responses [54]. Nonetheless, the process of schistosomiasis liver fibrosis is as complex as classical ecosystems, including cells, fibers, protein scaffolds, and chemical compounds [23]. Proliferation of hematopoietic cells (Fall-3^+^ cells and Sca-1^+^ cells) around hepatic vessels and around *S. mansoni* eggs trapped in the liver has been demonstrated [7]. The role of MDSCs in schistosomiasis liver fibrosis remains unclear.

The *S. japonicum* infection could alter the gut microbiota, which in turn might have effects on MDSCs. Our team previously reported the comparison of gut microbiota among HCs, CSJ, and AJS patients [12, 57, 58]. Fecal samples were collected and subjected to 16S rRNA sequencing analysis. These studies collectively found significant alterations in α and β diversity of gut microbiota with the progression of schistosomiasis. Specific taxa also showed varying relative abundances across disease stages. Notably, all three studies observed enrichment of *Prevotella* in the gut microbiota of CSJ patients compared to HCs, while its depletion was noted in ASJ patients. *Prevotella* might also be linked to NETs. Studies by Doke [5] and Palmer [30] found that *Prevotella Intermedia* associated with periodontitis could degrade NETs through nucleases release. Rangé identified *Prevotella Intermedia* in carotid atherosclerotic plaques, correlating with neutrophil activation/inflammatory markers [36]. Additionally, Neamah’s research in juvenile C57BL6 mice showed that tetrachlorodibenzo-p-dioxin activated Aryl hydrocarbon receptor, inducing massive accumulation of MDSCs along with *Prevotella* clustering in the gut [27]. Thus, changes in gut microbiota associated with *S. japonicum* infection may relate to MDSC accumulation.

It is well known that the immunopathological reaction in schistosomiasis is complex. Over the past several years, many studies have been devoted to the mechanism of immunopathology in schistosomiasis, especially the T-cell immune response [11]. Our study analyzed counts of T cells, B cells and NK cells in peripheral blood and their percentages in lymphocytes. At the same time, we analyzed the correlation between the absolute count of the above three cell types and the distribution of MDSCs and their subtypes among PBMCs. The findings revealed that the absolute count of CD4^+^ T cells correlated significantly and negatively with the distribution of G-MDSCs in peripheral blood.

In different types of schistosomes, eggs lead to different immunopathological characteristics, as manifested in different organs and pathological changes. For example, *Schistosoma haematobium* infection mainly leads to urinary system obstruction and inflammation, whereas infection by *S. mansoni* or *S. japonicum* infection mainly leads to liver and spleen inflammation, liver fibrosis, and intestinal diseases [11, 40]. In our study, schistosome eggs were not found in any *S. japonicum-* infected patient’s stool, which conformed to the positive rate of schistosome eggs in the Poyang Lake region of China [14, 49]. It indicated that due to the active control of *S. japonicum* and treatment with praziquantel, all the patients were not actively infected. Overall, liver ultrasound grading and serological indicators have certain value in the diagnosis of schistosomiasis in *S. japonicum* infection [28].

Interestingly, the absolute cell count of monocytes in peripheral blood and the percentage of monocytes among leukocytes were significantly different between the CSJ and ASJ groups, with both higher in the latter. This might be related to the Th2-type immune shift characteristic induced by parasitic infection, which leads to expansion of alternating activated macrophages [9, 37, 42], favoring the immunopathology of *S. mansoni* eggs [38]. We found that TBA, AST and ALB better reflected the different stages of schistosomiasis than levels of bilirubin. A recent liver biopsy study confirmed that the TBA-to-total cholesterol ratio (TBA/TC) can be used as a diagnostic indicator of the severity of liver fibrosis [50].

However, our research had the following limitations. First, the sample size was small, and the source of patients was mainly from part of the Dongting Lake Basin in China. Second, only the phenotype of MDSCs was detected, and effectors of MDSCs were not explored, and *in vitro* function assays were not performed. Compared to the chronic schistosomiasis group, the average absolute count of T cells in the advanced schistosomiasis group shows a decreasing trend, but the difference is not statistically significant, which may be related to the small sample size in the advanced schistosomiasis group. Finally, the mean age of HCs was lower than the schistosomiasis patients, which could have a potential impact on MDSCs.

## Conclusion

This study revealed that G-MDSCs in peripheral blood accumulate in humans infected with *S. japonicum*. The distribution and number of ^CD4+^ T cells in the peripheral blood correlated with accumulation of G-MDSCs, which might closely relate to impairment of host immune function and liver fibrosis.

## Data Availability

Data supporting the conclusions of this article are included within the article.

## Supplementary material

The supplementary material of this article can be found at https://www.parasite-journal.org/10.1051/parasite/2024050/olm.

*Supplementary Table 1*: Demographic information of the study population *Supplementary Table 2*: Clinical data for patients infected with *S. japonicum*
