# Supplementary material for: Proliferation of MDSCs may indicate a lower CD4+ T cell immune response in schistosomiasis japonica
Source: Parasite. 2024 Aug 29;31:52. doi: 10.1051/parasite/2024050 (PMC11363901; doi:10.1051/parasite/2024050)
Supplement: Supplementary file 1 — Supplementary Table 1: Demographic information of the study population [file parasite-31-52-s1.pdf]

Supplementary Table 1 The demographic information of the study population

	**Healthy controls (n = 21)**	**Chronic schistosomiasis japonica (n = 38)**	**Advanced schistosomiasis japonica (n = 18)**	**P value**
Male, n (%)	10, 47.62%	26, 68.42%	13, 72.22%	0.194
Age (Mean ± SD, y)	42.38 ± 11.60	59.50 ± 10.48	62.83 ± 12.63	< 0.001
