# Supplementary material for: Proliferation of MDSCs may indicate a lower CD4+ T cell immune response in schistosomiasis japonica
Source: Parasite. 2024 Aug 29;31:52. doi: 10.1051/parasite/2024050 (PMC11363901; doi:10.1051/parasite/2024050)
Supplement: Supplementary file 2 — Supplementary Table 2: Clinical data for patients infected with S. japonicum [file parasite-31-52-s2.pdf]

Supplementary Table 2 Clinical data for patients infected with *S. japonicum*.

**Clinical characteristics**	**All patients (n = 56)**	**Chronic schistosomiasis japonica (n = 38)**	**Advanced schistosomiasis japonica (n = 18)**	**P value** ▽
Splenic thickness (Mean ± SD) (mm)	33.63 ± 9.99	30.25 ± 5.65	43.10 ± 13.38	< 0.001 †
Length of the spleen under the ribs (Mean ± SD) (mm)	3.66 ± 11.03	0.00 ± 0.00	13.9 ± 18.50	< 0.001 †
Splenectomy, n (%)	6 (10.71%)	0 (0.00%)	6 (33.33%)	< 0.001 ╜
Inner diameter of the hepatic portal vein (Mean ± SD) (mm)	11.78 ± 1.30	11.41 ± 0.95	12.44 ± 1.59	0.010 †
abdominal dropsy, n (%)	7 (12.50%)	0 (0.00%)	7 (38.89%)	< 0.001 ╜
Depth of abdominal dropsy (Mean ± SD) (mm)	7.44 ± 23.11	0.00 ± 0.00	20.94 ± 35.58	0.003 †
Liver cirrhosis caused by *S. japonicum* infection, n (%)	12 (26.67%)	0 (0.00%)	12 (75.00%)	< 0.001 ╜
Portal hypertension by digestive endoscopy or ultrasonography, n (%)	3 (6.67%)	0 (0.00%)	3 (18.75%)	0.040 ╜
Long diameter of the left lobe of the liver (Mean ± SD) (mm)	70.45 ± 13.55	69.24 ± 9.64	72.77 ± 19.28	0.454 †
Anterior and posterior diameter of the left hepatic lobe (Mean ± SD) (mm)	55.29 ± 11.66	53.24 ± 12.76	59.23 ± 8.24	0.135 †
Maximum oblique diameter of the right lobe of the liver (Mean ± SD) (mm)	115.00 ± 13.17	115.90 ± 12.82	113.30 ± 14.20	0.575 †
Cavernous change or thrombosis of the portal vein, n (%)	2 (4.44%)	0 (0.00%)	2 (12.50%)	0.121 ╜
Hemoglobin concentration (HGB，g/L) (Mean ± SD)	134.80 ± 21.67	136.90 ± 18.70	130.90 ± 26.63	0.397 †
Eosinophils count (Mean ± SD) (10^9/L)	0.26 ± 0.29	0.25 ± 0.31	0.29 ± 0.28	0.625 †
Neutrophils count (Mean ± SD) (10^9/L)	3.47 ± 1.75	3.64 ± 1.67	3.15 ± 1.90	0.388 †
Lymphocyte count (Mean ± SD) (10^9/L)	1.72 ± 0.68	1.75 ± 0.56	1.67 ± 0.88	0.707 †
Platelet count (PLT，10^9/L) (Mean ± SD)	185.40 ± 71.07	194.50 ± 63.50	168.40 ± 83.06	0.256 †
γ-Glutamyl transpeptidase (γ-GT, U/L) (Mean ± SD)	51.25 ± 56.92	38.29 ± 44.19	76.00 ± 71.49	0.074 †
Total bilirubin concentration (TBIL, μmol/L) (Mean ± SD)	14.57 ± 6.20	14.23 ± 4.45	15.34 ± 9.21	0.583 †
Direct bilirubin concentration (DBIL, μmol/L) (Mean ± SD)	3.56 ± 2.46	3.18 ± 1.90	4.42 ± 3.36	0.118 †
Alanine aminotransferase concentration (ALT, U/L) (Mean ± SD)	25.00 ± 10.13	25.25 ± 9.66	24.43 ± 11.50	0.803 †
Globulin (GLO, g/L) (Mean ± SD)	25.90 ± 5.28	25.93 ± 5.11	25.82 ± 5.84	0.951 †

Fibrinogen (FIB, g/L) (Mean ± SD)	3.20 ± 1.02	3.39 ± 1.14	2.91 ± 0.76	0.255 †
International normalized ratio (INR) (Mean ± SD)	0.94 ± 0.11	0.92 ± 0.10	0.98 ± 0.13	0.266 †

▽: Comparison between patients with chronic schistosomiasis and advanced schistosomiasis. †: Tested by Unpaired t test. ╝: Tested by Fisher's exact test. ζ: Tested by Chi-square test.
